# Emerging MRI techniques for molecular and functional phenotyping of the diseased heart

**DOI:** 10.3389/fcvm.2022.1072828

**Published:** 2022-12-05

**Authors:** Hai-Ling Margaret Cheng

**Affiliations:** ^1^The Edward S. Rogers Sr. Department of Electrical & Computer Engineering, Institute of Biomedical Engineering, University of Toronto, Toronto, ON, Canada; ^2^Ted Rogers Centre for Heart Research, Translational Biology & Engineering Program, Toronto, ON, Canada

**Keywords:** heart failure, ischemic disease, non-ischemic disease, microvascular dysfunction, fibrosis, strain, metabolism, deep learning

## Abstract

Recent advances in cardiac MRI (CMR) capabilities have truly transformed its potential for deep phenotyping of the diseased heart. Long known for its unparalleled soft tissue contrast and excellent depiction of three-dimensional (3D) structure, CMR now boasts a range of unique capabilities for probing disease at the tissue and molecular level. We can look beyond coronary vessel blockages and detect vessel disease not visible on a structural level. We can assess if early fibrotic tissue is being laid down in between viable cardiac muscle cells. We can measure deformation of the heart wall to determine early presentation of stiffening. We can even assess how cardiomyocytes are utilizing energy, where abnormalities are often precursors to overt structural and functional deficits. Finally, with artificial intelligence gaining traction due to the high computing power available today, deep learning has proven itself a viable contender with traditional acceleration techniques for real-time CMR. In this review, we will survey five key emerging MRI techniques that have the potential to transform the CMR clinic and permit early detection and intervention. The emerging areas are: (1) imaging microvascular dysfunction, (2) imaging fibrosis, (3) imaging strain, (4) imaging early metabolic changes, and (5) deep learning for acceleration. Through a concerted effort to develop and translate these areas into the CMR clinic, we are committing ourselves to actualizing early diagnostics for the most intractable heart disease phenotypes.

## Introduction

Cardiovascular disease comes in many different forms, including ischemic disease associated with atherosclerosis, cardiomyopathies associated with aging and hypertension, arrhythmia, infection, valvular disease, and heart failure. Electrocardiography is a frontline diagnostic tool to detect abnormalities in heart rhythm, but deeper assessment always involves echocardiography to obtain real-time ultrasound images of the beating heart. From an echocardiogram, one can assess the structure and size of the heart wall and chambers, heart wall motion, valve morphology and function, and parameters related to cardiac output and ejection fraction. If echocardiography is performed in conjunction with a stress test (e.g., treadmill exercise), one can also probe how well the heart functions in response to stress.

While echocardiography permits a deep assessment of heart morphology and function, the indices measurable are symptoms of disease that has progressed for some time. Much more challenging to uncover are early indicators of disease development, or information at the tissue or molecular level. To probe at these deeper levels, one can resort to complementary three-dimensional (3D) cross-sectional imaging modalities that have functional imaging capabilities, such as nuclear medicine and magnetic resonance imaging (MRI). Nuclear medicine provides sensitive assessment of metabolism; MRI can also assess energetics while offering exquisite delineation of 3D structure and unparalleled tissue contrast. Similar to echocardiography, cardiac MRI (CMR) can delineate heart structure and measure chamber volume and wall thickness, and it can accomplish this with greater robustness owing to its 3D nature and high spatial resolution. CMR also provides information on wall motion, valve morphology and function, and cardiac output and ejection fraction. A distinctive power of CMR, however, is its unique ability to inform on tissue composition and microstructure. For example, the position and extent of infarcts are readily visualized on delayed contrast-enhanced CMR as a bright region devoid of cardiomyocytes and filled with a gadolinium-based MR contrast agent. However, today’s CMR goes far beyond infarct identification and delineation. Over the past decade, CMR has seen considerable technological development, strengthening its role as an indispensable complement to echocardiography for difficult phenotypes such as non-ischemic disease and heart failure with preserved ejection fraction (HFpEF), phenotypes that present abnormalities much more subtle than changes in gross structure or motion.

**FIGURE 1:**
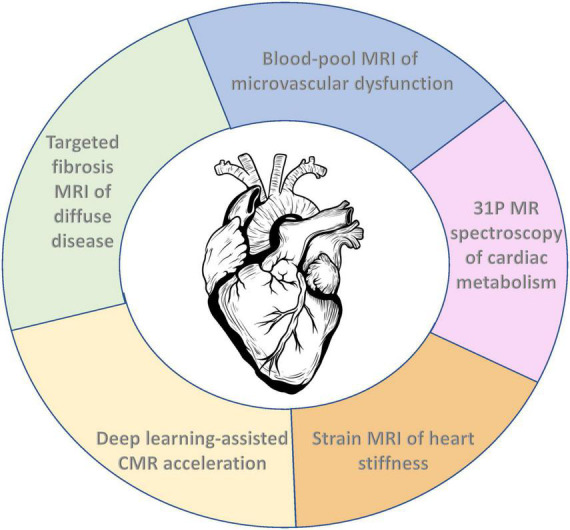
Emerging cardiac MRI (CMR) techniques for molecular and functional characterization of the diseased heart.

In this review, we will examine the latest CMR developments that enable us to probe at the microvascular, cellular, and molecular level. We begin with the most readily translatable and potentially most impactful technology, namely, new methods to interrogate microvascular dysfunction. While these are relatively nascent, they hold promise for a better understanding of certain cardiomyopathies and potentially early detection for at least half of all heart failure patients. Techniques to detect fibrosis are next discussed, given their value for detecting diffuse disease in non-ischemic settings, followed by strain CMR imaging of myocardial stiffness, another potential tool for early detection. We conclude with two technologies that are uniquely valuable but perhaps the furthest from clinical adoption: non-proton CMR to assess early metabolic abnormalities in heart failure, and artificial intelligence for accelerating CMR acquisitions (see Figure 1 for a depiction of emerging CMR techniques for phenotyping the diseased heart). Currently, none of these emerging CMR techniques is widely available in the clinic. However, with continued technological progress rooted in solving authentic clinical gaps, these emerging techniques may become standard CMR capabilities in the not-too-distant future.

## Imaging microvascular dysfunction

In contrast to atherosclerosis or other obstructive disease underlying many ischemic injuries, microvascular dysfunction (MVD) is a non-obstructive vessel disease (1). Microvessels function abnormally due to other reasons, such as ultrastructural vascular changes or aberrant response to stimulating factors. Not surprisingly, these changes can also reduce blood flow to the heart muscle, leading to ischemia. Another difference between obstructive disease and MVD is that the former is relatively straightforward to diagnose using angiography, whereas the latter is not, leaving many patients undiagnosed and untreated (2). To confirm MVD, the gold-standard approach is invasive right heart catheterization. Non-invasive alternatives also exist, such as stress tests involving MRI or positron emission tomography (PET). With these alternatives, a vasodilator is typically injected into the body to mimic microvascular response to exercise, and myocardial perfusion before and after injection are compared to identify regions with compromised reactivity. While stress perfusion testing has been a mainstay in this realm for decades, existing options for both the vasodilator and the injected contrast agent carry risks for certain patients (3). Newer options, as detailed below, have the potential to improve the safety, reliability, and specificity of MVD assessment compared to gadolinium-enhanced rest-stress perfusion CMR.

### Gas challenge as a vasoactive stimulus

**FIGURE 2:**
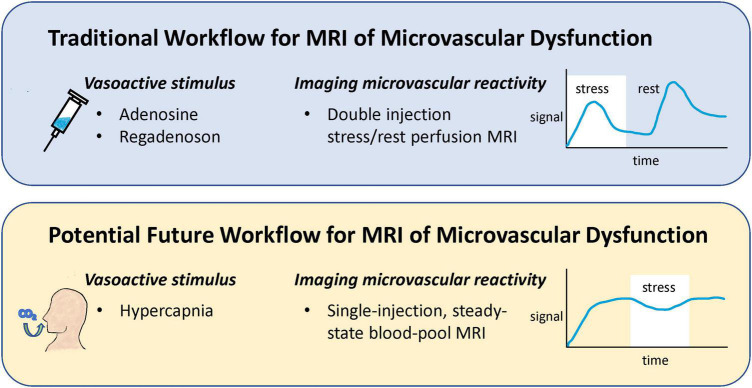
Comparison of a potential future workflow for MRI of microvascular dysfunction to conventional workflow. Instead of injecting a vasoactive drug to induce vasoreactivity, breathing elevated levels of CO_2_ is proposed as a safer, more repeatable vasoactive alternative. Furthermore, traditional assessment of perfusion change in response to stress via a double gadolinium injection protocol is replaced with a single-injection, blood-pool contrast imaging protocol that permits higher accuracy and specificity.

Current protocols for stress perfusion CMR involve administering a vasodilator, such as adenosine or regadenoson, to induce vasodilation of the coronary microvasculature and a double injection of a gadolinium-based contrast agent to measure perfusion both at stress and at rest (4). While generally safe, vasodilators carry the risk of severe hypotension, and some patients may require a second injection of a counteracting drug. Injecting a gadolinium chelate twice suffers from perfusion measurement bias for the second injection and from higher than usual gadolinium dosing. Recent research advances have proposed alternatives to both these shortcomings: altered inspired gas concentrations in place of adenosine or other pharmacological vasodilator (5), and use of a blood-pool contrast agent for single-injection assessment of microvascular reactivity (6) (Figure 2).

**FIGURE 3:**
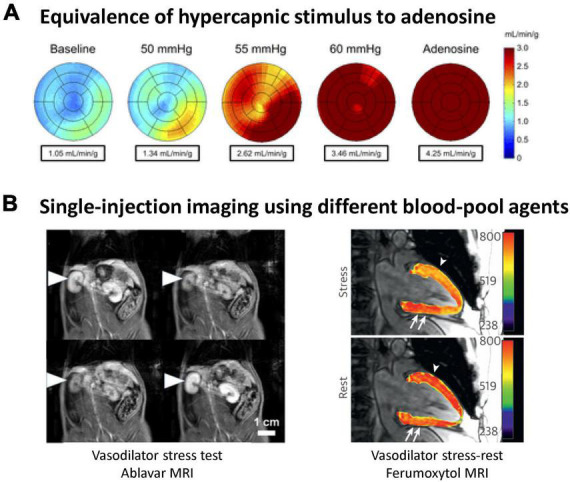
Illustration of hypercapnic vasoactive stimulus and single-injection, steady-state blood-pool imaging assessment of microvascular response. **(A)** Myocardial perfusion maps show the equivalency of gas challenge vs. pharmacological agents for inducing vasodilation. Global myocardial perfusion is shown in a representative subject at baseline, during hypercapnic stimuli, and during adenosine stress [this research was originally published in Pelletier-Galarneau et al. (9)]. **(B)** Blood-pool steady-state imaging for the assessment of microvascular reactivity. (Left) Vasodilation and vasoconstriction induced by a succession of hypercapnic maneuvers on a rat administered Ablavar. On T1-weighted images, the microvasculature of the renal cortex is seen to constrict (low contrast) on exposure to 20% CO_2_ but rapidly vasodilate (bright contrast) upon return to 5% CO_2_. [This research was originally published in Ganesh et al. (6). (Right) A patient with progressive angina presents a large, reversible inferior wall perfusion defect. Ferumoxytol-enhanced T_1_ map shows rest hypoperfusion in the inferior wall (arrows). At peak stress, ferumoxytol-enhanced T_1_ reactivity is 6% in the hypoperfused inferior wall (arrows) and 14% in the proximal anterior wall (arrowhead, net difference of 20%) [this research was originally published in Nguyen et al. (12)].

Inspiring elevated levels of carbon dioxide (CO_2_) is well-known for its vasoactive effects on the microcirculation. When inspired CO_2_ is elevated, a state known as hypercapnia, different vascular responses are seen, depending on the CO_2_ level and the tissue bed. At low levels of elevated CO_2_ (<5% inspired CO_2_) blood vessels in most tissue beds dilate, while higher levels evoke vasoconstriction in many organs (7). Compared to pharmacological agents, hypercapnia is safer, and its effects can be quickly reversed by restoring normoxia. Furthermore, a very reproducible response can be elicited. However, it was not until the introduction of a device for controlled gas delivery, the RespirAct™ system (Thornhill Research, Toronto, Canada), that hypercapnia was even a practical or reliable vasoactive stimulus for clinical use (5). This system is Health Canada approved and works by adjusting inhaled gas levels to target blood gas levels independent of minute ventilation or breathing pattern (i.e., little subject cooperation is required), thus making blood gas changes a robust, repeatable intervention. A large study of 434 exams in patients aged 9–88 confirmed the safety of this method (8). Most clinical research using the RespirAct has focused on neurological deficits, but some myocardial studies have emerged recently (9, 10). Perfusion increases in the myocardium from hypercapnia is comparable to that from pharmacological agents, as seen in Figure 3A in the healthy human heart, where hypercapnia induced a 3.5-fold increase and adenosine induced a 4.2-fold increase (9). Aside from targeting blood CO_2_ levels, the partial pressure of oxygen (pO_2_) can also be targeted to bring about hypoxic or hyperoxic effects on blood vessels, further bolstering one’s ability to assess both vasodilatory and vasoconstrictive response.

### Blood-pool imaging for assessment of microvascular reactivity

To detect inducible ischemia, where rest perfusion is normal, a stressor in the form of exercise or a vasoactive agent is needed to evaluate if certain parts of the myocardium are unable to increase perfusion adequately relative to healthy myocardium. The gold-standard for stress perfusion CMR is to inject a gadolinium chelate under maximally induced hyperemia and to measure perfusion during the first pass of the agent (11). The rest perfusion CMR can be acquired either before (4) or after (11) the vasodilator is administered. If the rest perfusion CMR is acquired after the stress perfusion CMR, a short interval after the vasodilator is administered [e.g., 10 min for adenosine (11)] must elapse before a second injection of gadolinium is given and first-pass perfusion again measured. A comparison of the stress perfusion and rest perfusion scans, even in a qualitative manner, then provides a clear depiction of areas affected by compromised vasoreactivity.

There are a few shortcomings with the gold-standard cardiac stress CMR described above. First, there is no room for error when it comes to contrast injection, and since there is only one window of opportunity to capture the first passage, the acquisition sequence and the contrast injection must be timed perfectly. Second, since the interval between the two injections is short relative to the elimination half-life of gadolinium chelate, an elevated baseline signal for the second injection will inevitably introduce errors in quantitative perfusion estimation. Even if T1 mapping is performed before each injection to convert signal intensity to gadolinium concentration, the pharmacokinetics is altered for the second injection due to residual contrast from the first. A final consideration is that the total contrast dose is double that of typical contrast-enhanced MRI exams.

Recently, the concept of blood-pool imaging of vasoreactivity was proposed to circumvent the limitations of conventional stress tests (6). A T1-reducing agent is introduced to sensitize T1 relaxation times to the intravascular compartment, as native T1 mapping is insensitive to blood volume changes from vasomodulation (7). A low dose of a gadolinium-based blood-pool agent, Ablavar^®^ (gadofosveset), is introduced intravenously and allowed to reach a quasi-steady state concentration after 10 min. This steady state lasts approximately 40 min in rats, even longer in humans due to a slower elimination rate. Over this time interval, one can administer a vasoactive substance, such as adenosine or hypercapnia, or even multiple stimuli in succession, and measure how much the microvascular blood volume changes. It is noteworthy that blood-pool imaging of vasoreactivity was initially conceived to permit a more specific and direct measure of vasomodulation (6). By looking at changes in blood volume, confounding factors—such as heart rate variations—implicit in conventional perfusion imaging are eliminated, thereby yielding a specific measurement of vasoreactivity. This approach also eliminates quantification errors resulting from double contrast injections. Several groups have since adopted the blood-pool imaging approach and applied it to myocardial stress testing (12, 13). Instead of Ablavar^®^, however, whose production was discontinued in 2017 due to low sales, these papers used Feraheme^®^ (ferumoxytol), an iron-based compound currently still approved in the U.S. as an iron supplement and used off-label as a blood-pool MRI contrast agent. Figure 3B illustrates blood-pool MRI of vasoreactivity in the kidneys, where blood-pool vasoreactivity imaging was first demonstrated, and the heart.

Blood-pool MRI contrast agents are valuable beyond their intended utility for angiography. They enable lower dosing and a much longer window for image acquisition. They also provide an alternative approach to assessing microvascular reactivity that permits higher specificity, quantitative accuracy, and spatial resolution than is possible with conventional double first-pass perfusion assessment of MVD. However, at the time of this writing, there is no clinically approved MRI blood-pool contrast agent on the market. Perhaps when more clinical indications in which blood-pool agents provide a clear advantage come to light, or when new gadolinium-free versions become accessible, there will be an incentive for pharmaceuticals to bring MRI blood-pool agents back into the clinic.

## Imaging fibrosis

**FIGURE 4:**
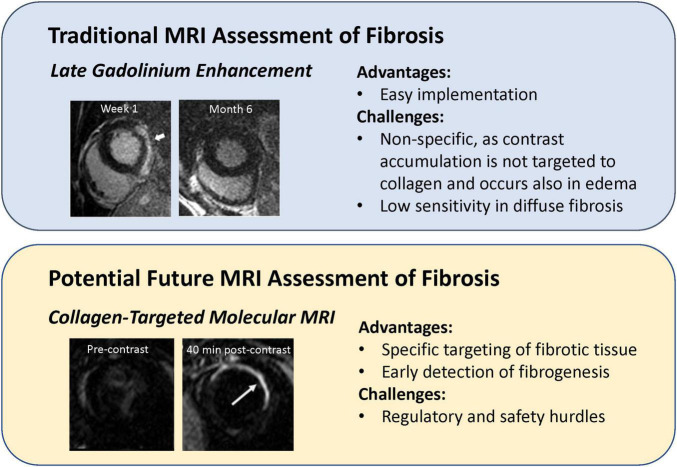
Comparison of targeted imaging of myocardial fibrosis to conventional late gadolinium enhancement. (**Upper panel**) Late gadolinium enhancement (LGE) in an acute setting does not mean definitive fibrosis, as LGE at 1 week after onset of acute myocarditis disappeared at 6-month follow-up [this research was originally published in Aquaro et al. (79)]. (**Lower panel**) Myocardial scar injected with a fibrosis-targeted agent, EP-3533, is not visible pre-contrast but is clearly visible 40 min after contrast injection [this research was originally published in Caravan et al. (16)].

Fibrosis is a progressive disease that affects virtually all life-sustaining organs and is seen in diseases of the heart, lungs, liver, and kidneys. In the setting of heart disease, fibrosis presents differently depending on the disease phenotype. For instance, in myocardial infarction, dead cells in the ischemic region are eventually replaced with scar tissue. This type of fibrosis, known as replacement fibrosis, is straightforward to identify with late gadolinium enhancement (LGE), because the cell-free infarct has much more space than healthy myocardium into which the contrast agent can diffuse. However, this technique works only in a mature infarct and is non-specific to collagen deposition. A case in point is an acute myocardial infarct where substantial enhancement is seen, but only edema is present as scar has not yet developed. Only at much later times when edematous fluids have resorbed and only scar tissue remains is LGE more clearly correlated with fibrotic tissue. A different type of scarring, one much more difficult to detect, is interstitial fibrosis. Here, collagen deposits are found between cardiomyocytes and in the perivascular space. Interstitial fibrosis can be of the reactive kind (associated with hypertension, diabetes, and renal insufficiency) or the much rarer infiltrative kind (associated with amyloidosis and Anderson-Fabry disease) (14). The current CMR method to evaluate interstitial fibrosis is extracellular volume estimation from a dynamic contrast-enhanced (DCE) MRI exam (15). The greater the extracellular volume, the greater the extent of fibrotic tissue assumed to occupy the interstitium. However, sufficient sensitivity is attained only when there is substantial fibrosis. Early detection of fibrotic tissue development, when collagen content is low but also when anti-fibrotic treatments would be best administered, remains an unresolved need. In the following, we describe novel targeted MRI contrast agents for imaging fibrotic tissue with higher specificity compared to conventional late-gadolinium enhancement (Figure 4).

### Collagen-targeted gadolinium-based contrast agents

In 2007, the first type I collagen-targeted MRI contrast agent, EP-3533, was introduced by Caravan et al. (16). The agent was a cyclic peptide with three gadolinium moieties, and the dense scar tissue of a mouse myocardial infarct was shown to enhance preferentially relative to surrounding healthy myocardium. Since the first demonstration in an infarction model, subsequent studies extended its application to mouse models of liver (17), pulmonary (18), and skeletal muscle (19) fibrosis. All studies confirmed superior sensitivity and specificity of collagen-targeted agents for fibrotic tissue compared to non-targeted counterparts. However, EP-3533 contained a linear gadolinium chelator used in DTPA, thus raising the risk of gadolinium toxicity. Recently, the collagen-targeting peptide in EP-3533 was attached to a more stable gadolinium macrocyclic chelate (20). Theoretically and in practice, macrocyclics are more stable than linear ones, and fewer reports of gadolinium deposition are associated with macrocyclic chelates. Nonetheless, because agents with an affinity to collagen, or any targeted agent, are designed to have longer residence time in the body than would a non-targeted agent, the safety profile must be conclusive before translation into humans is approved. Despite the fact that EP-3533 is currently not approved for clinical use, however, if its clinical translation is eventually successful, there would be a profound impact on disease detection, staging, and treatment monitoring.

### Non-gadolinium collagen-targeted contrast agents

Non-gadolinium agents targeted to collagen have been reported, but these are relatively few compared to EP-3533. For example, c(RGDyC)-USPIO is an iron oxide nanoparticle that targets liver fibrogenesis by imaging activated hepatic stellate cells that specifically engulf the contrast agent (21). Another example are manganese porphyrin-based contrast agents that have been used for labeling and tracking collagen scaffolds (22, 23) in tissue-engineering applications. These manganese agents offer very high sensitivity, and although they bound through non-specific mechanisms, it is possible to introduce new conjugation methods to specifically target collagen. The advantage of utilizing manganese-porphyrin is that manganese, unlike gadolinium, is a natural substance and is needed in small quantities by the body. Furthermore, the porphyrin ring provides a thermodynamically stable product such that demetalation *in vivo* is highly unlikely, even in long retention applications such as fibrosis imaging. Development of manganese porphyrin-based fibrosis contrast agents is currently geared toward both young scar (fibrogenesis) and old scar (fibrosis).

## Imaging strain

Strain imaging was introduced over a decade ago as a non-invasive echocardiographic method to assess myocardial stiffness and differentiate between passive and active movement of myocardial segments (24, 25). The objective was early detection of myocardial dysfunction before abnormal regional wall motion became obvious on conventional echocardiography. Compared to standard measures of ejection fraction (EF), myocardial strain is less dependent on heart rate and loading conditions and more sensitively detects subtle changes in the underlying substrate. In heart conditions such as HFpEF, chemotherapy-induced cardiotoxicity, and infiltrative diseases, strain imaging is a better predictor of outcome than EF, which may remain normal despite the presence of disease (26). Furthermore, while EF permits an indication of global heart function, objective measures such as strain rates overcome obvious limitations with regional wall motion assessment, which is subjective and depends on the experience of the reader.

### Strain cardiac MRI techniques

Cardiac MRI has also developed its own version of strain imaging. Several strain CMR techniques currently exist, but none is fully developed and standardization is a distant goal (27). Strain CMR was first introduced by Zerhouni et al. in which a magnetic label in the form of black lines was superimposed at the beginning of a dynamic CINE sequence and their deformation tracked through the cardiac cycle (28). This first strain imaging method is known as CMR tagging and is akin to speckle tracking in strain echocardiography (29). Both short- and long-axis images are acquired to measure motion in all three directions, which requires a longer series of breath-holds and is susceptible to image mis-registration errors. Furthermore, spatial and temporal resolutions are low and acquisition times long. Later methods for strain imaging were introduced to improve upon the capabilities of CMR tagging. Phase velocity mapping allows calculations of myocardial velocities in three directions and provides higher spatial resolution, albeit at the cost of lower temporal resolution (30). Displacement encoding with stimulated echoes (DENSE) encodes displacement in the phase image (31); while this technique inherently has low signal-to-noise (SNR), acquisition times are short. Finally, strain-encoded (SENC) imaging is similar to CMR tagging in that magnetization tags are used (32). However, rather than placing tags orthogonal to the imaging plane, SENC places tags parallel to the imaging plane, and only short-axis images are needed to measure longitudinal strain. Post-processing for SENC is quick, but one downside is that radial strain cannot be measured. Of all these strain CMR techniques, only CMR tagging has seen the most validation (33).

Feature tracking, unlike the methods described above, is a strictly post-processing technique and is applicable to any modality, not only MRI. No additional imaging is necessary, and standard CINE images can be retrospectively analyzed using feature tracking of cavity/myocardial borders to calculate strain. It is already available clinically and has demonstrated both reproducibility (34) and accurate delineation of infarct territories (35). However, as a contour-based tracking method, feature tracking cannot assess mid-wall strain and is limited by low spatial and temporal resolution, poorer performance on regional strain imaging, and lack of information on rotational strain (36). Therefore, despite its ease of use, feature tracking CMR tagging remains the accepted gold standard imaging modality for strain quantification.

### Clinical experience with strain cardiac MRI

Despite the lack of standardization for strain CMR, myocardial strain imaging has been investigated in different cardiac pathologies. Perhaps the most common application is ischemic heart disease. After a myocardial infarction, myocardial strain components are most impaired in the infarct (35) and strain analysis can identify which tissue segments will recover (37). However, given the availability of late gadolinium enhancement to identify infarct zones robustly, the role of strain CMR in this pathology is at best confirmative and not complementary.

A more difficult phenotype to diagnose is non-ischemic heart disease, where late gadolinium enhancement typically does not reveal evident tissue abnormalities. In this setting, strain CMR can be invaluable. Strain measurements have shown impairment in patients relative to healthy control subjects. For example, in hypertrophic cardiomyopathy, mid-wall fibrosis underlies abnormal systolic strain measurements with or without late gadolinium enhancement (38). Myocarditis also presents impaired myocardial strain in the absence of any other abnormality (39). Perhaps most notable is the potential for strain CMR to provide early indication of disease development. It has been demonstrated that abnormal alterations in strain parameters manifest much earlier than reductions in ejection fractions, hypertrophy, or even presentation of symptoms (40). It is for this reason that strain imaging is routinely recommended in patients undergoing chemotherapy, who are at risk for cardiotoxicity from chemical agents like doxorubicin.

### The landscape for strain cardiac MRI vs. strain echocardiography

**FIGURE 5:**
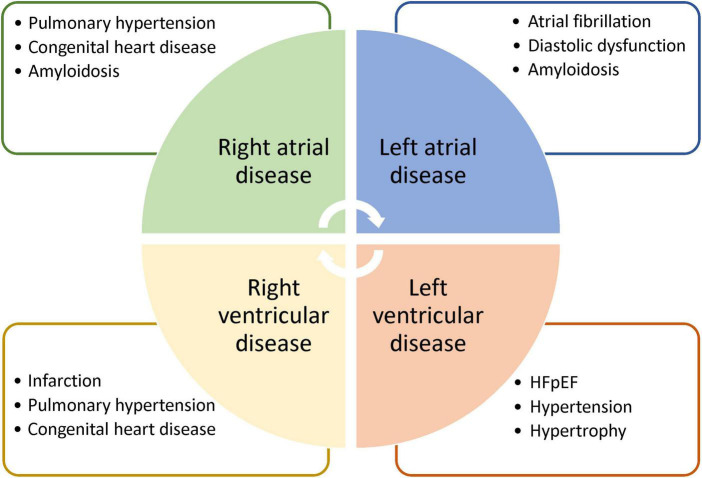
Potential applications of strain cardiac MRI. Strain MRI is suggested for deeper phenotyping of diseases affecting different heart chambers. Note that each list is intended to highlight potential applications and may contain diseases that are not exclusive to a particular chamber.

Strain echocardiography is a validated technique for assessing subtle myocardial changes. However, it is used strictly for larger muscular structures. It is unsuitable for thin structures of the right ventricle and the two atria. Strain CMR, on the other hand, is the gold-standard for assessment of the right ventricle and is the first modality to demonstrate right ventricular strain analysis (41). Diseases of the right ventricle, while less common than those of the left ventricle, affect a significant patient population: those living with congenital heart disease, pulmonary hypertension, or arrhythmogenic right ventricular cardiomyopathy. Similarly, atrial strain measurement is only possible on CMR. Essentially, deformation of the atrial walls can become aberrant in diseases characterized by high filling pressures and diastolic dysfunction. Pathologies exhibiting these properties include HFpEF (42) and myocarditis (43), where atrial strain CMR have demonstrated promising results. Given the utility of strain CMR in characterizing not only pathologies that strain echocardiography can detect but also those that echocardiography cannot, a very real need exists to bring about consensus amongst MRI vendors to standardize strain CMR and ensure quality control. Figure 5 illustrates a few potential applications for strain CMR.

## Imaging early metabolic changes

Heart disease frequently involves early changes in cell metabolism before overt structural and functional alterations set in. The concept of using non-proton MR spectroscopy to assess the heart is not new. In fact, a study in 1979 already described the utility of 31-phosphorus (31P) MR spectroscopy in assessing adenosine triphosphate (ATP) levels in living hearts and those that had undergone potassium chloride-induced cardiac arrest (44). Fast-forward forty years, and this type of metabolic imaging remains rare in the clinic. However, recent studies suggest a resurgence of interest and value across multiple heart disease phenotypes. In the sections below, we focus specifically on 31P MR spectroscopy owing to its growing utility that has been demonstrated in the clinical setting. Other nuclei also exist for MR spectroscopy, such as hyperpolarized 13-carbon and 17-oxygen. However, these remain primarily studied in preclinical settings; the reader is referred to many excellent reviews on these topics.

### Cardiac 31-phosphorus spectroscopy

13P MR spectroscopy assesses muscle energy metabolism by monitoring the ratio of phosphocreatine (PCr) to ATP. Cardiac muscle is like any other muscle in our body in that it requires a substantial supply of ATP to power contractions. When an ATP molecule is hydrolyzed to release energy, it produces adenosine diphosphate (ADP) and an inorganic phosphate as by-products. To maintain a constant energy reservoir, ATP must be regenerated; the molecule PCr is required to transfer high-energy phosphate to convert ADP into ATP. During a period of low energy requirement, excess ATP is used to convert creatine back to PCr. Therefore, PCr acts as a high-energy reserve to maintain normal ATP levels regardless of fluxes in energy demands. Since ATP levels are relatively stable, assessment of the muscle cell’s energy store is achieved by monitoring the ratio of PCr and the three phosphate groups on ATP. Any decrease in the PCr/ATP ratio is indicative of a depleting PCr store.

### Indications for cardiac 31-phosphorus spectroscopy

Heart failure is a prime example of a disease phenotype where 31P MR spectroscopy is informative. The healthy heart forms ATP by oxidizing both fats and glucose, and the proportion from each substrate is adapted to the environment—fatty acids are the major substrate in the healthy and trained heart, whereas glucose utilization increases in heart failure. Regardless, utilization of both substrates is reduced in the failing heart, resulting in a decline in the PCr/ATP ratio (45). This observation has been confirmed in both human studies of advanced heart failure [e.g., dilated cardiomyopathy (46)] and induced heart failure in mice (47). Patients with a confirmed HFpEF diagnosis also present a lower PCr/ATP ratio, approximately a 25% decrease, compared to healthy control subjects (48). Perhaps even more illuminating is that the comorbidities associated with HFpEF—such as diabetes (49) or the typical hypertrophic phenotype of HFpEF (50)—present similar reductions in the PCr/ATP ratio. The discriminatory power of imaging energetics extends to subclinical cardiac dysfunction—obese patients demonstrate a modest but significant decrease in PCr/ATP at rest, a decrease that is further exacerbated during a dobutamine stress test (51). These examples illustrate that clinical implementation of cardiac 13P MR spectroscopy, while complicated, is not impossible.

### The promise and hurdles of clinical 31-phosphorus spectroscopy

The most significant limitation of 31P MR spectroscopy is the low sensitivity of the 31P nucleus. The gyromagnetic ratio of 31P, at 17.2 MHz/Tesla, is 40% that of the hydrogen proton. Furthermore, phosphate metabolites exist at relatively low concentrations in the body. Therefore, to attain adequate SNR, voxels tend to be large and acquisitions prohibitively long for clinical implementation. In recent years, increased use of higher field magnets has translated to higher SNR, which can be traded for shorter acquisitions or better spatial resolution. More significantly, several acceleration methods have been proposed for 31P MR spectroscopy. Echo-planar spectroscopic imaging, which exploits rapidly oscillating readout gradients to encode both spectral and spatial information, was demonstrated for 31P MR spectroscopy in the human calf muscle with a scan time of 10 min (52). A spiral version of the oscillating gradient has also been demonstrated for muscle 31P MR spectroscopy (53). Despite a much faster scan time, however, both techniques incurred an SNR penalty. Perhaps more interesting is a subspace technique called SPICE (for SPectroscopic Imaging by exploiting spatiospectral CorrElation) introduced in 2014 for rapid, high resolution proton spectroscopy (54). The same group later applied SPICE to 31P MR spectroscopy, wherein they achieved a resolution of 6.9 × 6.9 × 10 mm^3^ and 15 min scan time when imaging human calf muscle on a 3 Tesla scanner, with the spatial and temporal resolution improving to 1.5 × 1.5 × 1.6 mm^3^ and 30 frames/s, respectively, at 9.4 Tesla (55).

**FIGURE 6:**
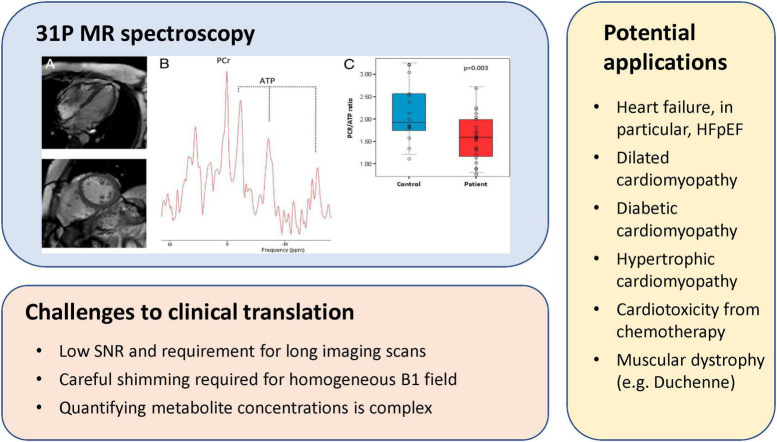
31P MR spectroscopy of myocardial energetics, challenges, and potential applications. (**Upper left panel**) CMR images of a patient with heart failure with preserved ejection fraction (HFpEF), corresponding localized 31P MR spectra from the left ventricle, and individual PCr/gamma-ATP ratio in patients with HFpEF and in control subjects [this research was originally published in Phan et al. (48)].

Other challenges also hamper clinical translation. 31P MR spectroscopy is inherently complex, but obtaining a good 31P spectrum of the heart is even less trivial, even if on a qualitative level. Both cardiac and respiratory motion complicate acquisition, and acquisition time is prolonged due to the need for triggering. Obtaining a homogenous B1 field is also important but is time-consuming and often difficult. Furthermore, qualitative assessment of PCr/ATP is suboptimal in the context of detecting early disease, as shifts in metabolism may not be yet quite pronounced. A better approach would be quantitative measurement of absolute PCr and ATP levels. While more tedious, one can achieve quantitation by including a reference phantom outside the body. Continued development of cardiac 31P MR spectroscopy toward clinical adoption will need to see advances in high-SNR coil design, rapid imaging, dynamic shimming, and greater access to higher field strength scanners. All of these will improve spectral and spatial resolution and reduce acquisition times. With continued technological advances in these areas, metabolic imaging will one day be part of routine clinical workup for cardiac patients. Figure 6 illustrates a 31P MR spectroscopy experiment, existing challenges to clinical translation, and applications that may benefit greatly from this technology.

## Deep learning for acceleration

The application of deep learning to medical imaging has seen an exponential growth over the past decade. The ultimate vision is to have deep learning assist in diagnosis, but the earliest endeavors targeted more tangible objectives such as image segmentation (56, 57). Even the most recent effort focused on image reconstruction from highly undersampled data (58, 59) is more straightforward than medical diagnostics. To date, success in deep learning-assisted acceleration has been reported in many types of MRI acquisitions, including cardiac. Acceleration in CMR can potentially reveal disease phenotypes currently difficult to probe, such as abnormalities in diastolic function (still best assessed on echocardiography) that appear in patients with diabetes and other metabolic syndromes, or mechanical abnormalities that are most prominent during an irregular heartbeat. In the following sections, we describe the most notable advances and the outstanding challenges in this domain.

### Deep learning strategies for accelerated MRI

The premise for acceleration can be formulated as an optimization problem, where the original high-resolution image must be reconstructed from a sub-Nyquist undersampling of the acquisition domain, namely, k-space. The original strategies would provide aliased images (aliased due to severe undersampling) to a neural network and train against ground-truth unaliased images from fully sampled datasets (60). The neural network is essentially tasked with learning the Fourier transformation to convert images from the image domain to k-space and vice versa, as well as learning features of the training dataset. Variations on this image-to-image approach also exist. For example, one can feed in undersampled k-space datasets and train against fully sampled unaliased images (61), or one can feed in undersampled k-space datasets and train against fully sampled k-space (62). For the latter approach, the neural network learns how to fill in missing k-space datapoints.

Most acceleration applications to date exploit the convolutional neural network architecture (63). This choice is fitting for network applications involving images, because images can be more sparsely represented as a summation of two-dimensional kernels representing edges in different orientations, different shapes, and any “building-block” structure. Different layers of the neural network represent features at different scaling factors. The deeper the layer, the coarser the feature. More recently, a novel architecture has been investigated with great interest. This architecture, known as the transformer (64), differs from the convolutional neural network in that it is based on a concept called attention and is ideally suited to handling sequential tasks while capturing long-range dependencies. Originally conceived for natural language processing, where one needs to look at dependencies between widely separated words to construe the meaning of a sentence, the concept of attention can also be extended to an image, where pixels far apart but contributing to the same structure must be recognized as an intact entity. Applications of the transformer to image denoising, motion deblurring, and defocus deblurring have been reported (65). In the area of image reconstruction alone, transformers have demonstrated superior performance compared to conventional convolution neural networks (66, 67).

Adaptions of the reconstruction techniques described above can involve, as examples, training on both the image domain and k-space or getting the neural network to uncover the optimal undersampling pattern in k-space. The first method, also known as dual-domain reconstruction, makes the reasonable and logical assumption that providing both object and frequency domain data for training should improve reconstruction quality (67, 68). While this is, indeed, the finding, it also comes to no surprise that the improvement in reconstruction quality is modest, since the information contained in k-space is identical, no more and no less, than the information contained in the image. The second approach of identifying the optimal sampling region of k-space invariably results in an oversampled center of k-space and sparser sampling in the peripheral spatial frequency regions (69). This behavior is also expected. Perhaps a useful piece of information that can be gleaned from these studies is the ideal probability distribution of k-space sampling. Most, if not all, of these sampling patterns cannot be realized upon implementation, but they can inform on how to best sample k-space in a statistical manner given real constraints on gradients.

### Limitations of acceleration through deep learning

Despite the successes of different neural network architectures in achieving high quality reconstruction from undersampled acquisition data, there are quite a few technological shortcomings that should be recognized. Most obvious is the availability of large datasets for training. There are a handful of MRI databases currently available, such as the UK Biobank, which contains the largest collection to date of acquisitions in various body regions. For instance, it has an abundant repository of different types of brain and cardiac images. Assuming that access to a large database is not an issue, the next hurdle, one much more difficult to overcome, is the high dependency of training on the acquisition itself. If the tissue type changes, or any of the acquisition parameters (e.g., imaging plane orientation, spatial resolution, image contrast, field-of-view, sequence type, and vendor type) changes, the network must be re-trained for the new scenario. It is easy to appreciate that this limitation can severely curtail the clinical utility of deep learning-assisted acceleration.

**FIGURE 7:**
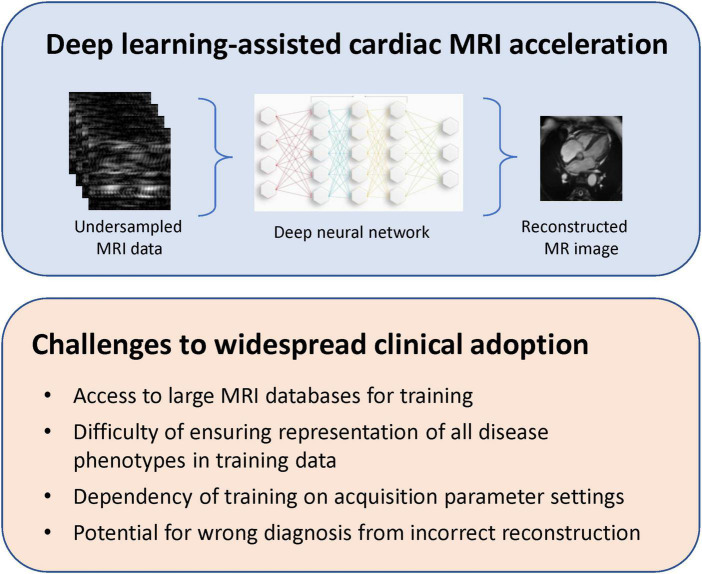
Deep learning-assisted cardiac MRI acceleration. Despite having demonstrated an ability to reconstruct high-quality cardiac MRI from undersampled k-space datasets, deep learning-assisted MRI acceleration face important challenges that must be addressed to ensure flexible, reliable, and widespread adoption.

Recently, researchers have proposed so-called physics-based deep learning methods to partially address some of the limitations described above. These are dubbed physics-based, because the neural network is trained to solve a supervised learning task and, at the same time, adhere to any physical laws governing the system of interest. As an example, rather than treating each image as a new reconstruction problem, we can exploit known information in regard to the anatomy of interest or aliasing artifacts specific to different k-space trajectories. In the first physics-based reconstructions, the undersampling pattern and coil sensitivities, both with known effects on the reconstructed image, were taken into account to solve an inverse problem (59, 70). However, these applications employed fully sampled data as a reference during training. In cardiac imaging or myocardial perfusions scans, where it may be infeasible to have fully sampled training datasets, one is compelled to train on undersampled data. In 2021, Yaman et al. (71) introduced a self-supervised deep learning approach to train physics-guided reconstruction without fully sampled reference data. While their platform was demonstrated on brain and knee data, it would be instructive to extend their network to cardiac MRI. Figure 7 presents a schematic of a deep learning-assisted reconstruction of undersampled MRI data and existing challenges that must be overcome to permit widespread clinical adoption.

## Discussion

Cardiac MRI is an excellent adjunct to echocardiography in providing information on heart anatomy and mechanical function. It also provides unique tissue characterization, such as delineating the extent of an infarct. Yet, in this review article, we have seen that emerging CMR techniques can explore cardiac tissue and function on much deeper levels, from structurally intact but functionally abnormal microvessels to pathological shifts in metabolic substrates. The question remains as to whether deeper phenotyping is necessary and beneficial. What types of heart disease can deeper phenotyping benefit, and how can it impact patient care? Heart failure with preserved ejection, which affects over half of all heart failure patients, is a difficult-to-treat disease that can benefit immensely. It is a heterogeneous disease with multiple subtypes spanning multiple comorbidities (hypertension, obesity, diabetes, and kidney disease). Treatment will likely require a tailored approach, namely, fine-tuned characterization at the diagnostic level. For example, imaging microvascular dysfunction may provide early detection before the HFpEF heart takes on the classical signs of stiffness and hypertrophy. Alternatively, metabolic imaging with 31P MR spectroscopy may distinguish between different subtypes brought on by obesity vs. hypertension. A second heart disease phenotype that can benefit from deeper phenotyping is that involving diffuse fibrosis. For example, patients with hypertension are predisposed to developing myocardial fibrosis; yet, this tissue alteration is very difficult to detect on conventional cardiac imaging until the late stages of disease. If a fibrosis-targeted MRI scan can diagnose fibrogenesis at its early stages, then there is hope of intervening to slow or reverse progression. It is important to emphasize that while we can already envision what disease phenotypes will benefit from a deeper, more specific characterization of the heart, the full value of the technologies described in this review can only be appreciated when they are readily available, easily accessible to all heart patients.

All the emerging techniques discussed in this article are well-established in both physics-based and preclinical studies. To facilitate their full clinical translation for early diagnosis of difficult heart disease phenotypes, we need to address outstanding impediments. In the following, we will examine each of the five emerging CMR techniques and identify both remaining hurdles and realistic solutions.

Assessing coronary microvascular dysfunction is a decades-old practice, but it is also one in which the MRI version of stress testing is fraught with confounders that render accurate quantitation quite difficult. Obtaining two accurate absolute perfusion measurements (at rest and at stress, or vice versa) in quick succession by injecting two full doses of a gadolinium chelate is all but impossible. Residual contrast from the first dose, unless given sufficient time to clear from the body, will interfere with the pharmacokinetics of the second injection dose. The only practical solution is to employ a single injection that maintains a relatively stable blood profile over the tens of minutes of a stress test—only a blood-pool contrast agent can fulfill this role. Unfortunately, since 2017, there has not been a single MR blood-pool contrast agent approved clinically for imaging. The only one currently available on the market, ferumoxytol, is used off-label in the U.S. and is not approved in Europe or Canada. Low market demand was cited as the reason for stopping the production and distribution of Ablavar in 2017, despite the radiological benefits it provided to pediatric cardiac patients. Another possible reason for its retraction, although never confirmed, was potential concern for gadolinium demetalation from a long-circulating blood-pool chelate with an elimination half-life of 16 h. A possible solution is to develop blood-pool MR contrast agents that have a non-gadolinium MR-active metal. Manganese is a viable alternative, with five unpaired electrons as opposed to seven for gadolinium. By binding manganese with a porphyrin, a very stable structure can be exploited for blood-pool MR imaging (72, 73). While the feasibility and safety of these newer MR blood-pool agents have been demonstrated, it is up to the MR physics and radiology community to engage with big pharmaceuticals (e.g., Bayer) and convince them that there is a real need for safer MR contrast agents with versatile functionality.

Current assessment of fibrosis is non-specific, because a measured increase in the distribution volume for gadolinium accumulation may or may not be related to collagen deposition. For nearly 20 years, researchers have described strategies to target gadolinium-based contrast agents to collagen, the protein that is greatly elevated in fibrotic tissue. Despite ample preclinical evidence of sensitivity and specificity, none of these molecular contrast agents have gained access to the clinical space. A plausible explanation is that with gadolinium-based agents, the chance for demetalation is simply too great for any application requiring prolonged retention in the body, and the risks to patients unacceptably high. Similar to blood-pool MR contrast agents, these targeted molecular agents would also benefit from utilizing a non-gadolinium MR-active metal. A fertile direction for further research is non-gadolinium MR contrast agent design and development, and this needs to happen in both the research lab and in industry.

The landscape for strain CMR is competitive, as strain echocardiography has demonstrated immense power in recent years for early left ventricular disease diagnosis and even prognosis. An equivalent capability from any other modality, including strain CMR, would face stiff competition from a gold-standard that is portable, inexpensive, and widely available. However, because delineation of the atrial and right ventricular walls is challenging on echocardiography, these are the applications in which strain CMR can make a real impact. The greatest existing hurdle to widespread adoption of strain CMR is repeatability. Although high reproducibility of global strain measurements has been demonstrated for different types of strain CMR approaches (74–76), reproducibility for more difficult segmental strain analysis is just emerging (77). If vendors can come to a consensus and provide standardized, reproducible strain CMR platforms, multiple diagnostic opportunities will emerge. Examples include measuring left atrial strain to assess risk of atrial fibrillation and left ventricular diastolic function and filling pressure, measuring right atrial strain to assess pulmonary hypertension, and measuring right ventricular strain to assess right ventricle infarction or pulmonary hypertension.

Metabolic imaging using 31P MR spectroscopy holds promise for very early detection of abnormal utilization of energy substrates in a variety of cardiac diseases, including heart failure, dilated and hypertrophic cardiomyopathies, hypertensive heart disease, coronary artery disease, and type I and II diabetes. The main technical limitation of 31P MR spectroscopy is its intrinsically low SNR. Moving to a higher field strength, such as 7 Tesla scanners, is one solution—in one report, the SNR gain was used to shorten to acquisition from 31 min on a 3 Tesla system to 6 min at 7 Tesla (78). These high-field systems remain rare in the clinic. Taking 31P MR spectroscopy down the translation pathway would need to see advances made in parallel along two fronts: first, there needs to be buy-in from vendors to manufacture more high-field scanners and at a lower price; second, there must be more reproducibility studies demonstrating the sensitivity and specificity of 31P MR spectroscopy across different heart pathologies.

Real-time or near real-time CMR using deep learning is a rapidly evolving field. If successful, we can image cardiac dynamics as it happens in real-time, akin to echocardiography. However, its main Achilles’ heel is 2-fold: need for large training datasets and the possibility of incorrectly reconstructing image features on which the network has not been trained. The first hurdle is surmountable for some types of MRI applications, including cardiac, but access to large databases will inevitably become a roadblock, unless existing algorithms can generalize beyond fixed acquisition parameter settings. The second hurdle is a much more difficult ethical question to address. How can one ensure that all disease phenotypes that can ever be encountered are available for training? Furthermore, since artifacts from reconstruction can bear remarkable resemblance to real pathology, how can radiologists be guided in distinguishing real from fake image features? The simple answer is that deep learning can never enter clinical decision making if it can lead to a false positive or, worse yet, false negative diagnosis. Notwithstanding this drawback, a better view of how deep learning can be clinically useful is to apply it to situations to which the patient would otherwise not have access. For example, if one could generate a deep-learning perfusion scan from a very low dose injection, then a diabetic heart failure patient who cannot take the full dose due to risks of gadolinium would not be deprived of the benefit of a contrast-enhanced exam. With creativity around how we use deep learning, it can become a very intelligent agent to the radiologist.

## Author contributions

H-LC drafted and edited the manuscript.
